# Digital undergraduate medical education and patient and carer involvement: a rapid systematic review of current practice

**DOI:** 10.1186/s12909-023-04218-z

**Published:** 2023-05-16

**Authors:** Sadie Lawes-Wickwar, Eitan Lovat, Adedoyin Alao, Julia Hamer-Hunt, Nesrin Yurtoglu, Cherise Jensen, Nicola Clarke, Nia Roberts, Sophie Park

**Affiliations:** 1 Department of Primary Care & Population Health, University College London, London, UK; 2 School of Medical Education, Newcastle University Medical School, Newcastle Upon Tyne, UK; 3 Department of Psychiatry, University of Oxford, Oxford, UK; 4 Bodleian Health Care Libraries, Medical Sciences, University of Oxford, Oxford, UK

**Keywords:** Medical education, Digital healthcare, Patient and public involvement, Remote learning

## Abstract

**Background:**

Involving patients and carers in medical students’ learning aims to centralise the perspective of healthcare users and supports our future medical workforce in the development of key skills. Medical schools are increasingly using digital technology for teaching and it is timely to understand how to maintain patient and carer involvement in this context.

**Methods:**

Ovid MEDLINE, Ovid EMBASE and medRxiv were searched in October 2020 and reference lists of key articles were hand searched. Eligible studies reported authentic patient or carer involvement in undergraduate medical education where technology was also used. Study quality was assessed by the Mixed Methods Appraisal Tool (MMAT). Levels of patient or carer involvement were assessed using Towle et al.’s (2010) taxonomy, from Level 1 (lowest level) to Level 6 (highest level).

**Results:**

Twenty studies were included in this systematic review. In 70% of studies, patients and carers featured in video or web-based case scenarios with no interaction between healthcare users and students. The remaining 30% of studies reported real-time interactions between students and patients via remote clinical encounters. Digital teaching sessions involving patients or carers were perceived to be valuable by students and educators, and increased student engagement, patient-centred attitudes, clinical knowledge, and communication skills. No studies reported the perspective of patients or carers.

**Discussion:**

Digital technology has not yet driven higher levels of patient and carer involvement in medical training. “Live” interactions between students and patients are becoming more common but challenges need addressing to ensure positive experiences for all involved. Future teaching should enhance the role of patients and carers in medical education and support them to overcome any potential barriers to doing so remotely.

**Supplementary Information:**

The online version contains supplementary material available at 10.1186/s12909-023-04218-z.

## Background

Patient and public involvement (PPI) in medical education includes teaching, assessment, feedback, and curriculum development [1]. Global consensus is that medical school stakeholders should be partners in the education of the future medical workforce [2] and this is a requirement of professional regulatory bodies. For example, in the UK, PPI is a requirement of the General Medical Council (GMC) [1]. In early 2020, the Covid-19 pandemic forced medical centres globally to reduce face-to-face contact, and remote teaching became the standard approach for medical schools. This reduced opportunities for interactions between patients and students. With the benefits of PPI in medical teaching wide-ranging, including developing students’ person-centred skills [3], improving students’ professional attitudes and clinical performance, and offering professional, personal, and emotional benefits for healthcare users [3, 4], continued PPI in the era of online learning is crucial. Involving patients and carers in remote medical education is also timely and will help students respond to the evolving needs of patient groups as remote healthcare delivery continues [5]. Developing remote consultation skills will undoubtedly be a key requirement for the future medical workforce [6, 7].

A number of challenges arise from this new way of working. Firstly, patient and carer “involvement” varies widely, with implications for remote teaching practice. A recent taxonomy of involvement defines active PPI in medical teaching as a spectrum, from featuring in case studies (“Level 1”) to involvement at an institutional level, e.g. in decision-making (“Level 6”) [8]. Historically, patients and carers have held relatively passive roles in the education of medical students, but examples of good practice have increased over recent years [9]. However, these examples are from in-person teaching and other than electronic case studies and pre-recorded patient videos [8–10], the variety of potential uses of digital technology in medical training when patients and carers are also involved has not been explored. A recent scoping review of PPI in rural healthcare education settings found patients had been involved over telephone and in online materials, in consultations about new curricula and evaluating programmes [11]. This initial insight however needs to be expanded to all medical education contexts (including other geographical areas) to inform the future strategy of medical schools globally.

There may be barriers for patients and carers invited to join medical teaching sessions and research from traditional (face-to-face) medical training has found these include a lack of knowledge about medical education [12] having a sensitive clinical problem and concerns about privacy or confidentiality [12, 13]. However, findings from digital healthcare allude to new barriers introduced when joining remote healthcare consultations. For example, according to one study, patients report being unable to access the necessary technology, and may find connecting remotely more difficult due to their symptoms [14]. For marginalised patient groups remote healthcare may exacerbate language barriers and reduce opportunities for practical support from reception staff such as registering and signposting [15]. We must first understand the specific barriers that may limit PPI in remote medical education, to ensure medical training is inclusive of diverse voices, and representative of local populations.

Two further recent systematic reviews have described active PPI in medical education broadly [3, 16], however these reviews were not focused on the use of technology for learning. The aims of the present review therefore were to present the variety of digital technologies that have been used in medical teaching when patients and/or carers are also involved, and what has been the experience of patients, students and educators alike.

## Methods

Rapid systematic review methods were employed. Rapid reviews follow standard systematic review procedures, whilst providing timely evidence and maintaining rigour [17]. Rapid methods were chosen to provide teaching teams with timely evidence for the uses of technology to support continued PPI in undergraduate medical education after the rapid shift to remote working during the COVID-19 pandemic.

### Protocol

The protocol has been registered on PROSPERO Ref. CRD42021243279. The review protocol is available on this PROSPERO web page.

### Search strategy and selection criteria

Searches for published and unpublished studies were performed from database inception to 27^th^ October 2020 using MEDLINE (OvidSP), EMBASE (OvidSP) and medRXiv Preprints (https://www.medrxiv.org/) by a university librarian (NR). The search strategy is available as a supplementary file (Supplementary File 1). Boolean and proximity operators were used, for example digital*. Searches were not limited by language or publication date. Retrieved references were initially de-duplicated in Endnote before being exported into Rayyan [18] and titles and abstracts were screened by seven authors (SLW, AA, JHH, NY, CJ, NC & SP). Ten percent of titles and abstracts were screened independently by two authors (AA & NC) and any disagreements were discussed with a third author (SLW) until consensus was reached. This was limited to 10% of articles due to time restrictions.

Primary studies evaluating undergraduate medical education activities were eligible. Eligible studies also described any type of digital technology, including remote technology (e.g. telephone, video-conferencing software), or technology used in-person that could be adapted for remote use (e.g. video). Studies involving patients and/or carers at any level [8], and employing any study design, were eligible. Eligible studies also reported student-, educator- and/or patient-related outcome data. Studies explicitly describing the use of actors (without experience of the medical problem they were presenting with) or other persons not presenting as authentic patients or carers were excluded. Non-English language articles were excluded at screening stage, due to the rapid nature of this review and a lack of resources to translate studies. Attempts were made to retrieve articles from the authors’ institutions but if unsuccessful the article was excluded, due to time and funding restrictions. Reviews were excluded, but reference lists were hand searched for additional studies.

### Data extraction and analysis

A data extraction form was developed by the authors based on the Sample, Phenomenon of Interest, Design, Evaluation, Research type (SPIDER) criteria, developed for reviewing qualitative and mixed methods studies [19]. Data extraction was completed by 7 authors independently (SLW, AA, NC, JHH, NY, EL, CJ), all extracted data was reviewed by SLW for completeness. All student-, educator- and/or patient-related outcome data was extracted, as well as the type of technology, demographics of involved patients and/or carers, types and levels of PPI, and study design. Due to the heterogeneity of study designs, a narrative synthesis was performed. A taxonomy of active PPI in healthcare education [8] was used to categorise the level of patient and/or carer involvement in the educational activity described by study authors. Categories range from patients being involved in developing a case study/ scenario—but had no overall influence on the theme of the content, nor on curriculum development (Level 1)—to patients being involved at the institutional level (Level 6) [8].

### Quality assessment

The Mixed Methods Appraisal Tool (MMAT [20]) was used to assess study quality. The MMAT has been used for most common study methodologies and in a variety of contexts including health sciences, education, information sciences and psychology [20]. Two authors were independently involved in the appraisal process (EL & NC); double assessment was not performed due to time limitations. MMAT scores were categorised as low, moderate or high-quality using criteria employed for two recent rapid systematic reviews of public health interventions [21, 22]; a score of 0–1 was categorised as low quality, 2–3 moderate quality, and 4–5 high quality.

### Patient and Public Involvement (PPI) in the research team

The review team included two public contributors (JHH, NY), who joined the team at the stage of planning the review (after the research question had already been defined). Both public contributors had specific experiences as patients, of literature reviewing, and as PPI representatives on research teams at Oxford University (JHH) and University College London (NY), as well as experiences contributing to medical education. One public contributor also had lived experience as a carer. JHH and NY were members of the research team, joined research meetings, and supported the review processes including literature screening, data extraction and interpretation, and preparing the manuscript for publication. PPI contributors informed decisions about our inclusion criteria, ensuring the review considered the carer viewpoint.

## Results

### Study selection

**Fig. 1:**
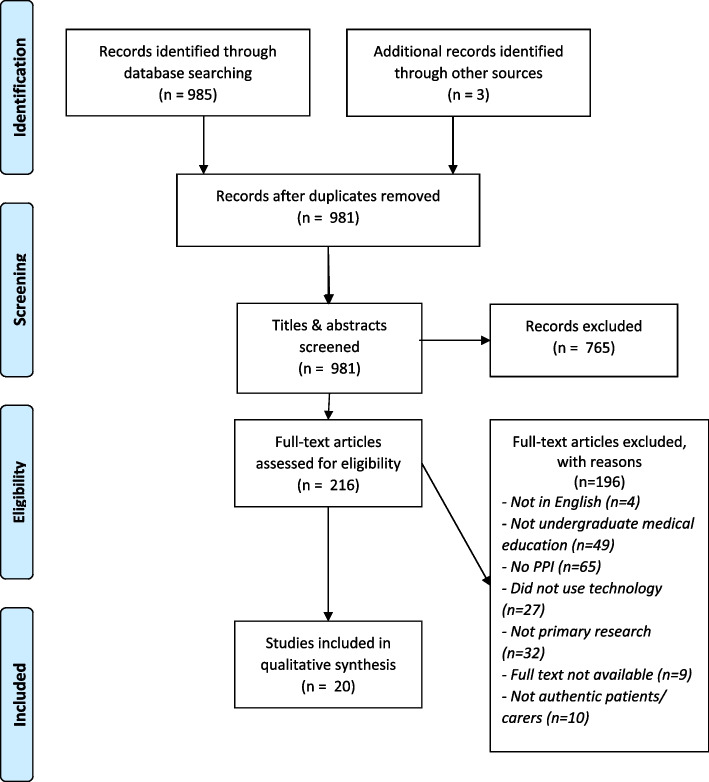
PRISMA Flow Chart

The full texts of 216 potentially relevant articles were screened for eligibility. A total of 20 studies were identified as eligible and included in the review (Fig. 1).

### Study characteristics

Characteristics of the articles included in the review and the types of digital technology used to support educational interventions involving patients and carers are presented in Table 1.

**Table 1:** Characteristics of included studies

**Authors, year, country**	**Study Design**	**Sample size (n)**	**Description of educational activity and study aims**	**Technology used**	**Description of involved patients/ carers and their role**	**Description of study participants (students, educators, patients)**
**Carson et al. 2020, USA **[23]	Single arm pre-post study; Quantitative research	17 students	GP-patient consultations were pre-recorded and then shown to year 1 medical students to establish the educational value of demonstrating clinical interactions to them in this way	Video	Real patients featured in videos	**Students:** Fourth-year medical students
**Colonnello et al. 2020, Italy **[24]	Cross-sectional survey; Quantitative research	130 students	Medical students watched two video clips of patients undergoing surgery. One of the videos had a narrative describing the patient’s emotional state before the video was played. The aim was to establish if incorporating such information enhanced students’ attitudes towards educational videos and whether it was educationally advantageous in terms of learning surgical techniques	Videos from online medical education platforms	Real patients undergoing surgery featured in videos	**Students:** second year medical students, 72F, 58 M
**Coret et al. 2018, Canada **[25]	RCT; mixed quantitative/qualitative research	27 students	First year medical students were randomly assigned to either watch a video lecture about patients with intellectual and developmental disabilities (IDD) or watch the lecture and subsequently watch reflective discussion of videos featuring people with IDD. The experimental group also then participated in an interactive patient simulation. The aim of the study was to assess the benefits of using a blended learning experience	Videos taken from the online Curriculum of Caring “Voices of experience” series (https://machealth.ca/https://machealth.ca/)	Real patients featured in videos of patient discussing their experiences of hospital and interactions with clinicians	**Students:** First-year medical students (23F, 4 M)
**D'Alessandro et al. 2004, USA **[26]	Longitudinal survey; Quantitative research	79 medical students	Patients and family experiences of common paediatric problems were transformed into a holistic digital story-line. These were then uploaded to the internet for anyone to access, including medical students. The aim of the study was to assess the benefits of using patient-centred computer based learning	Computer-based digital story-telling scenarios	Real patients and their families experiencing a common paediatric problem featured in the digital scenarios via interviews, photographs and growth-charts	**Students:** 79 medical students (other non-medical students also involved in the study but their results are not being considered)
**Darnton et al. 2020, UK **[27]	Cross sectional study; Qualitative research	13 students, 10 GP tutors	As a result of Covid19, student-patient interaction in the primary care setting was restricted. The study aimed to assess the acceptability and ease of remote consulting. Students took part in 3 remote clinics with patients (supervised by GPs)	Remote consultations (AccuRx, Microsoft teams and telephone)	Real “live” patient encounters over remote technology	**Students:** second year graduate-entry medical students (9F, 4 M) **Doctors:** 7 GP practices linked with the University of Cambridge medical school (6F, 4 M; 6 were GP practice partners; 5 were approved to supervise trainees)
**Dow et al. 2020, UK **[28]	Cross sectional survey study; Qualitative research	11 students in the focus group; 162 students gave feedback forms	Medical students staffed a Covid19 hotline and provided patient-counselling and education telephonically. They learned audio-only examination skills, clinical decision making skills and triaging skills. Their comfort level with undertaking these tasks was assessed retrospectively using a pre- and post-assessment	Panopto ® Video platform	Real patient clinical interactions featured in videos	**Students:** First-year medical students
**Fog-Petersen et al. 2020, Denmark **[29]	Longitudinal ethnographical study; Qualitative research	12 students	Students were given access to a video library of interviews with psychiatric patients by a doctor, along with the MSE report. The aim of the study was to assess students use of the video library to supplement their learning	A video-library of psychiatric-patient/doctor interactions	Real patient clinical interactions featured in videos	**Students:** fifth year medical students (7F, 5 M)
**Gorniewicz et al. 2013, USA **[30]	RCT; Quantitative	18 students	Semi-structured interviews were conducted with patients suffering from cancer. Themes emerging from these interviews, relating to cancer-care, were used to develop educational training modules. Students were also shown snippets from these interviews as part of the module. The aim was to test the effectiveness of such a model to deliver breaking-bad news training	Video	Real patients and family members featured in videos	**Students:** 18 medical students (the demographics for medical students is not broken down further; other study participants are excluded from this systematic review)
**Harless et al. 1990, USA **[31]	Single-arm pre-post study; Quantitative research	306 students	An interactive videodisc was created and then presented to a classroom. The lecturer (in consort with the medical students) could verbally interact with the videodisc as though it was a real patient, thus playing the role of physician. The aim of the study was to assess the believability of such a simulation and whether students learned essential knowledge as a result of the learning experience	Video	Professional actors and non-actors with experience of the health problems they were portraying in their patient roles featured in the video	**Students:** second-year medical students
**Kindratt et al. 2019, USA **[32]	Single arm pre-post study; Quantitative research	28 students	Students participated in a blended learning experience (online diadactic training, classroom based session and clinical examinations) to enhance their ability to promote early literacy and school readiness when doing well-child visits. The online training included four modules: (1) benefits of Reach Out and Read; (2) ways to incorporate books in children’s lives; (3) teachable moments/techniques for clinical settings; and (4) research	Online diadactic training	Online training was based on a national training programme featuring real patients in videos (unclear if real patients featured in the adapted version)	**Students:** 28 medical students (out of a group of 94 students overall) *Further demographics are reported but are not broken down to separate medical from non-medical students*
**Klemenc-Ketis et al. 2013, Slovenia **[33]	Cross-sectional survey study; Quantitative research	147 students	Students completed a 3-h problem-based learning assignment at the end of the seventh semester (Year 4). Virtual clinical cases are used for students to make clinical decisions (interventions, referrals etc.). The clinical cases were taken from a freely available e-forum, moderated by a specialist in family medicine, and where registered patients can submit questions. The medical educators chose the appropriate virtual clinical cases for the students to use in their assignment	Online medical forum (“Med.Over.Net”)	Real patients submitted clinical problems in an e-forum	**Students:** Fourth-year medical students (95F; 52 M)
**Knight et al. 2016, Australia **[34]	Mixed methods study; Qualitative/quantitative	150 medical students; 10 medical practices in New South Wales, Australia	Telehealth technology was used to help encourage consultations between patients in a primary care with a specialist consultant. In addition to providing benefits to the patient, the aim of the study was to evaluate the clinical and educational outcomes of this experience by medical students	Telehealth remote technology	Real “live” patient encounters over remote technology	**Students:** 150 medical students from University of Wollongong **Doctors:** 9 medical practices in rural areas and one in an urban-based Aboriginal setting
**Leeds et al. 2020, USA **[35]	Cross-sectional survey, Quantitative	54 students	Medical students were shown a 13-min, patient-centred narrative video entitled “Fibromyalgia: A Patient’s Perspective (FPP)” featuring patients suffering from fibromyalgia	Video	Patient narratives informed the video content (unclear if real patients featured in video)	**Students:** Third-year medical students 29F, 23 M
**Newcomb et al. 2020, USA **[36]	Cross-sectional survey, Qualitative	5 students	Fourth year medical students attended a 2-h class virtually where they learned skills about building rapport and trust with patients and their families. They subsequently practiced these skills with simulated patients. The aim of the study was to pilot a class for teaching video-based communication skills	Video conferencing software (Zoom)	"Simulated patients” with actor training (unclear if real patients) featured “live” in role plays during remote class	**Students:** fourth year medical students
**Shapiro et al. 2009, USA **[37]	Cross-sectional survey; mixed quantitative/qualitative	32 students	Students filmed their encounters with patients during home-visits over a period of 8 months and subsequently edited the film-material down to a 10 min presentation. They presented their presentation to an audience of students and faculty	Video	Real patients featured in videos	**Students:** 10 first-year medical students, 22 s-year medical students (18F, 14 M, aged between 22–30)
**Smith et al. 2011, USA **[38]	RCT; Quantitative research	199 students	Students were shown a video of a simulated female Arab-American-Muslim patient receiving care by a white-male GP with the aim of improving their cross-cultural sensitivity	Online interactive patient-simulation	Real patients and Arab American Muslim community members took part in focus groups on which simulation content was based	**Students:** second year medical students (99F, 100 M; Caucasian *n* = 156, Asian-American *n* = 24 other ethnic groups *n* = 19
**Snow et al. 2016, UK **[39]	RCT	88 medical students	Students were randomised to either watch a video of a consultant describing a colposcopy, or patients describing their colposcopy. The aim of the study was to assess if providing students with the patients-perspective had positive outcomes on examination performance	Video	Authentic patients who had previously undergone a colposcopy described their experience of having a colposcopy	**Students:** fifth year medical students
**Sweeney et al. 2018, UK **[40]	Single arm pre-post study; Quantitative	48 students	Videos of patients discussing their hospital experiences were shown to a group of medical students. Students then had discussions, in small groups, around themes of communication and patient experience	Videos	Real patients featured in videos	**Students:** medical students at the end of their first year of clinical placements (third-year medical students)
**Weber et al. 2020, USA **[41]	Cross sectional feasibility study; Mixed quantitative/qualitative research	64 students	A four-week virtual elective was designed to allow students to continue with patient interactions virtually as a result of reduced patient contact because of the Covid19 pandemic. The aim was to ensure satisfactory completion of curricular competencies	Telephone and video conferencing (“Doxy.me”, Facetime)	Real “live” patient encounters over remote technology	**Students:** fourth-year medical students
**Yoon et al. 2016, South Korea **[42]	Cross sectional survey; Quantitative research	99 students	Students had a video-based problem based learning (PBL) experience. They subsequently underwent a PBL experience involving a simulated patient. The aim of the study was to compare the use of simulated patients PBL to video-cased PBL	Video-recordings	Unclear if real patients were involved in videos or as “simulated patients”	**Students:** second-year medical students

#### Types of technology used

Six of the final 20 studies (30%) used remote healthcare and remote learning technologies, including telehealth platforms and video-conferencing software, to engage live with patients. Telehealth platforms were used for live remote clinical consultations [27, 28, 34, 41]. One class was delivered via video-conferencing software where “simulated” patients were featured in role plays with students [36], and one class involved an online blended learning module [32]. The remaining 14 studies (70%) used pre-recordings and existing online materials featuring patients, where there were no live interactions between students and real-time patients. Three studies used existing online patient materials to facilitate in-person teaching sessions [26, 33, 38], e.g. an online medical e-forum with clinical questions previously submitted by patients [33]. Eleven studies used pre-recorded videos to provide the patient’s perspective on their illness or demonstrate doctor-patient consultations [23–25, 29–31, 35, 37, 39, 40, 42] during in person teaching sessions.

#### Description of patients and carers involved in medical education

Two studies described using “simulated” or “virtual” patients, [36, 42] and two studies used patient-focused videos [32, 35], but none of these three studies clarified these terms, or whether patients were authentic. Sixteen studies involved authentic patients [23–31, 33, 34, 38, 40, 41] and two studies included the perspectives of family members [30, 32]. One of these two studies did not provide enough detail to determine if caregivers were authentic carers of patients, or whether they were actors [32].

#### Levels of patient and public involvement (PPI) in medical education

In the majority of studies (*n* = 14), students viewed a pre-recorded video or completed online material that involved no interaction with patients and were thus categorised at Level 1 of Towle and colleagues’ [8] taxonomy [23–26, 29–33, 35, 37–40, 42]. Six studies involved patients in real-time clinical encounters led or observed by students [27, 28, 34, 41], reflecting Level 2 of Towle et al.’s taxonomy, although in two of these studies the authenticity of the patients is unclear [23–26, 29–33, 35, 36, 38, 42]. No study reported patient and/or carer involvement above Level 2.

### Quality assessment

The MMAT score distribution for the included studies were summarised as follows: low quality *n* = 3 papers [25, 31, 33], moderate quality *n* = 2 [22, 40] and high quality *n* = 15 papers [23, 24, 26–30, 32, 34–38, 41, 42]. The two mixed-methods studies scored 5/5 and 3/5 respectively in the quality criteria for their qualitative and quantitative components respectively [23, 32].

### Synthesis of results

A summary of the main results is reported in Table 2. The results have been synthesised below in relation to the impact of digital educational activities involving patients and/or carers, on medical students, educators, and patients/ carers themselves.

**Table 2:** Results of the included studies

Authors, year, country	PPI/ technology mode	Patient outcomes	Student outcomes – Acceptability & attitude towards activity	Student outcomes – Attitude towards patients/ perceptions of involvement	Student outcomes – Knowledge of condition, treatment, patient group	Student outcomes – Clinical & communication skills	Educator outcomes	Study quality
**Darnton et al. 2020, UK **[27]	**Authentic—LIVE**	Remote consulting was acceptable to patients (according to the interviewees)	(1) Students were satisfied with no travel time required, and less "dead time" however some had to go to great lengths to ensure the environment was suitable (2) There was a general anxiety about technology failure among both supervisors and students. Observing students/doctors were uneasy about stepping in when there were glitches to confirm what had been said / might have been missed in the "down-time" (3) Mixed views on educational value—most felt it was beneficial, but some thought it was second-best. However others thought it was an essential skill due to the evolving nature of medicine as a result of the pandemic	Not reported	Not reported	More difficult to develop a good patient rapport	(1) Supervisors were satisfied with how students had set up and maintained an appropriate environment for the consult (2) Supervisors found it more difficult to built rapport with students, making it harder to give feedback (3) Supervisors found it more difficult to do a pre- and post- consultation chat with students (4) Remote consulting resulted in reduced informal contact between supervisor/students—this made it harder to give critical feedback for some students	High
**Knight et al. 2016, Australia **[34]		Not reported	Students commented that their involvement enhanced their learning	Not reported	The experience allowed students to appreciate the difference in consulting practices between GPs and hospital consultants. There were also benefits in terms of learning about tele-psychiatry, and enhancing job readiness	Not reported	Not reported	High
**Weber et al. 2020, USA **[41]		Most patients from 222 encounters specifically requested transition to a traditional phone call (101, 46.5%) or reported inability to access a compatible smartphone and/or computer at the time (71, 32.7%). Despite assistance from students, 40 (18.4%) patients were not able to enter the virtual waiting room	Students felt that interviewing patients, collecting the history and documenting the encounter provided significant educational value to themselves	Not reported	Not reported	Not reported	Not reported	Low
**Newcomb et al. 2020, USA **[36]	**“Simulated”—LIVE**	Not reported	Not reported	Not reported	Not reported	Students felt they developed new skills and reinformed current skills including self-confidence in exploring patient’s perceptions, sharing information with patients and checking understanding with patients. Students were particularly appreciative of the opportunity for direct observation of their communication skills and the ability to receive immediate faculty feedback	Not reported	High
**Carson et al. 2020, USA **[23]	**Authentic – RECORDED (video)**	Not reported	Not reported	Not reported	Not reported	Students reported increased comfort with (a) answering questions related to Covid-19 (*p* = 0.006) and screening patients for Covid19 (*p* = 0.0446) (b) assessing exam findings over the phone (*p* = 0.0429) (c) triaging patients (*p* = 0.0103) and (d) addressing rural financial challenges (*p* = 0.0127)	Not reported	Low
**Colonnello et al. 2020, Italy **[24]		Not reported	Incorporating patient’s emotional state into surgical videos enhanced students’ engagement with the video (*p* = 0.02) and motivation to watch the video again (*p* < 0.001)	Not reported	Not reported	Not reported	Not reported	Moderate
**Coret et al. 2018, Canada **[25]		Not reported	All students thought that the blended educational experience including video narratives of and direct interactions with people affected by IDD was valuable and enjoyable	Not reported	Not reported	Students had higher mean communication performance scores across all patient educator interview stations when they had received the blended educational activity versus the control activity (completing a quiz in a standard lecture setting). Students involved in the blended educational experience reported greater self-rated measures of confidence, and competence compared to control;	Not reported	High
**D'Alessandro et al. 2004, USA **[26]		Not reported	Students found the digital story-telling system to be of educational value. 98.8% of medical students felt they would be able to evaluate a similar patient problem and 91% felt they would remember at least some aspect of the digital stories in the future. Some students, however, would have appreciated more interactivity	Not reported	Not reported	Not reported	Not reported	Moderate
**Fog-Petersen et al. 2020, Denmark **[29]		Not reported	The video library helped compensate for the limited amount of patient contact. However, shortcomings included not being able to question the Professors MSE assessment when they disagreed with it and not being correct by their supervisor when their MSE contained mistakes	Not reported	Not reported	Not reported	Not reported	High
**Harless et al. 1990, USA **[31]		Not reported	Students felt emotionally and intellectually involved and challenged by the patient simulation video cases. The majority of students indicated support for the use of the video-simulated patient case in their education. The video-simulated patients were perceived as acceptability by students – they preferred this method to traditional lectures	The patients featured in the video-simulations were perceived as realistic	Students learned core clinical content by watching the Technological Innovations in Medical Education (TIME) videos. Significant gain in knowledge (*p* < 0.02) of “essential clinical content”	Not reported	Not reported	High
**Shapiro et al. 2009, USA **[37]		Not reported	Students rated the project highly for its impact on their education. Student viewers found the films compelling and informative	Not reported	Student filmmakers reported learning about the impact of chronic illnesses on relationships, the psychological impact of chronic illnesses, the roles of allied health professionals, the availability (or lack thereof) of some community resources, and, to a lesser degree, about insurance challenges, adherence issues, and the financial impact of care	Not reported	Faculty members also found the student-films compelling and informative	High
**Snow et al. 2016, UK **[39]		Not reported	Not reported	Not reported	No statistical difference in self-reported improvement of knowledge about cervical cancer screening between the two groups	Students in the experimental arm scored higher in the OSCE examinations, were more confident in their understanding of how to communicate with patients about cervical screening, were more comfortable in discussing cervical cancer screening with patients and were also more comfortable responding to patients emotional reactions after viewing video featuring patients	Not reported	High
**Dow et al. 2020, UK **[28]		Not reported	93% of students felt the experience was educationally valuable. They also appreciated watching patient-doctor consultations in a non-simulated, realistic set up	Not reported	Not reported	Focus-group students felt the videos helped them understand how to apply their learning to real-life medicine, how they need to adapt their history taking skills to meet the 10-min time pressure and how to vary their approach based on patient needs	This method worked well for both the GPs recording the consultation and the teaching team—the facilitated discussions with students were enthusiastic and interactive	High
**Sweeney et al. 2018, UK **[40]		Not reported	Not reported	There was an improvement in patient-practitioner orientation scores (indicating an improvement in patient-centred attitudes) by students after watching videos of patient discussing their hospital experiences and interactions with clinicians	Not reported	Students reported changes in their approach to patients, including introducing themselves more often, and taking measures to make patients feel more at ease on ward rounds	Not reported	High
**Klemenc-Ketis et al. 2013, Slovenia **[33]	**Authentic – RECORDED (Case studies)**	Not reported	Not reported	Not reported	Not reported	Not reported	Factor analysis of new assessment tool to evaluate students’ communication skills. Student’s can only be reliably assessed by a single assessor	High
**Leeds et al. 2020, USA **[35]		Not reported	87% of students felt the video was helpful to learners and 79% felt it was superior to a lecture	Students’ attitudes towards fibromyalgia and patients with fibromyalgia increased significantly from pre-video topost-video (*P* < .0001). Post-video students were more likely to report empathy for patients with fibromyalgia, as well as positive feelings about treating them in the future	There was a significant improvement in the student knowledge of fibromyalgia (*p* < 0.0001) after watching the educational video “Fibromyalgia: A Patient’s Perspective”	Not reported	Not reported	High
**Smith et al. 2011, USA **[38]		Not reported	Not reported	Not reported	Students who watched the interactive patient simulation had improved diversity knowledge, cultural sensitivity and cross-cultural comfort than those participants in the control arm of the study	Participants who were exposed to an online educational tool reported greater self-efficacy in ability to communicate with Arab Americans than participants in the control condition	Not reported	Low
**Yoon et al. 2016, South Korea **[42]	**“Simulated” – RECORDED (video)**	Not reported	Students showed improved motivation scores from interacting with standardised patients than using video materials (*p* < 0.001)	Significantly higher scores for attitude towards patients (*p* < 0.001) in problem-based learning using simulated patients than using video	Not reported	Students perceived simulated patients led to significantly better collaborative learning, (*p* < 0.01), reflective thinking (*p* < 0.001) and patient-doctor communication (*p* < 0.001) in problem-based learning than using video	Not reported	High

#### Impact on medical students’ learning and attitudes

Nineteen of the 20 articles reported the impact of PPI via digital tools on students’ learning and attitudes. Two of these 19 studies included mixed samples of medical students, residents [30, 32] and nursing and pharmacy students [30] where the outcomes for medical students from other healthcare students could not be extracted, so their findings have not been reported below. The remaining 17 studies measured student-reported outcomes of PPI on their learning when this was combined with the use of technology. These included acceptability, attitudes towards the activity, attitudes towards patients and/or carers, clinical knowledge and communication skills. Two of these 17 studies included objective measures of students’ learning, e.g. interpersonal skills (scored by a blinded simulated patient) [39].

##### Acceptability and general attitudes towards educational activity

Six studies found digital activities involving patients and/or carers to be educationally valuable [25, 26, 28, 31, 40, 41]. Two high quality studies reported that students found the educational activity acceptable [31, 34]. One high quality qualitative study investigating student-led remote consultations reported mixed student perceptions about the educational value and acceptability of these remote interactions with patients, with some reporting a preference for in-person consultations (e.g. due to being unable to perform a physical examination), while others found the experience valuable [27].

In one high quality study students reported positive attitudes towards video libraries featuring authentic patient cases [29]. Another high quality study found 79% of students reported that a 13 min video of a patient’s perspective of fibromyalgia was superior to a traditional in-person lecture [35].

##### Attitudes towards patients

Three out of 20 articles reported positive students attitudes towards patients after digital activities involving patients or carers. Two of these studies found improvement in students’ patient-centred attitudes after watching videos of patients discussing their condition or hospital experiences [23, 35]. Yoon and colleagues, however, reported traditional problem-based learning led to significantly improved attitude towards patients, compared to videos of patient cases [42]. Although, notably, it is unclear if patients were authentic in either the standardised or video-delivered approach in this study.

##### Knowledge of condition or treatment featuring in educational activity

Out of six articles reporting students’ clinical knowledge or knowledge about the patient group featuring in digital activities, five reported gains in students’ knowledge [31, 34, 35, 37, 38]. One high quality study reported no differences in self-reported knowledge about cervical screening when students viewed a video involving patients, versus a video featuring a clinician [39].

##### Clinical and communication skills

Six studies reported improvements in students’ communication skills after a digital activity involving patients and/or carers. One qualitative study reported a remote class with “simulated” patients helped students develop skills in exploring patient’s perceptions, sharing information with patients, and checking their understanding [36]. An online educational tool featuring a Muslim woman was found to improve students’ self-efficacy in communication with Arab American patients than participants in the control condition [38]. Dow and colleagues [28] reported videos helped students understand how to adapt their history taking skills and vary their approach to meet patients’ needs. Coret and colleagues [25] reported higher communication scores after a blended learning activity (with online elements) versus a standard lecture. Students reported introducing themselves more often, and taking measures to make patients feel more at ease, after watching videos of patients discussing their hospital experiences [40]. Snow et al. [39] reported higher OSCE scores, more confidence communicating with patients, and students feeling more comfortable responding to patients’ emotional needs, after watching a video of patients sharing their experiences of colposcopy, compared to a video featuring a clinician only. One low quality study found a student-led clinical hotline for patients with COVID-19 increased students’ remote clinical skills in screening, assessment, and triaging patients [23].

The traditional patient simulation was found significantly more beneficial to students in their collaborative learning, reflective thinking, and patient-doctor communication, than a video-delivered simulation in the study by Yoon and colleagues [42]. Further, student-led remote consultations were reported by some students to inhibit rapport-building with patients versus in-person consultations [27].

#### Perspective of medical educators

Only four of the 20 included articles reported the perspective of medical educators of digital educational activities involving patients and/or carers.

##### Acceptability and value of educational activity

Video-recorded GP consultations featuring patients were reported to facilitate discussions with students [28]. Tutors found student-made films about the impact of living with chronic conditions (with PPI) to be compelling and informative [37]. While GP supervisors were satisfied with some aspects of student-led remote consultations, including how students set up and maintained appropriate environments for consultations, the physical distance made it difficult to build rapport with students, with fewer opportunities to offer students feedback [27].

##### Perceptions of students’ skills

One high quality study reported that an e-forum for patients was a suitable learning tool for tutors to assess students’ clinical decision-making skills [33].

#### Perspective of patients and/ or carers

##### Acceptability of educational activity

Darnton and colleagues [27] reported that student-led remote consultations were acceptable to patients, but this was from the perspective of students and educators. No studies measured the acceptability of PPI in students’ learning via digital technology from the perspective of patients or carers involved.

##### Barriers to participating in educational activity

Weber and colleagues [41] reported difficulties for patients attempting to participate in telehealth consultations led by students. Out of 222 encounters, 46.5% of patients requested a traditional telephone call (over the telehealth consultation), 32.7% reported not having access to a compatible smartphone and/or computer and 18.4% had difficulty with the technology and were unable to join the virtual waiting room [41].

## Discussion

### Main findings

The aim of this rapid systematic review was to identify the uses of, and evaluate, digital technology in undergraduate medical teaching when patients and/or carers have been involved, encompassing all educational settings, technologies and geographical locations. Twenty articles met the eligibility criteria and demonstrated a variety of potential uses of digital technology in undergraduate medical education when patients and carers are involved.

The review found that PPI was perceived to be educationally valuable to students and educators, acceptable to students, and increased students’ knowledge of patient groups, as well as communication and clinical skills. Limited evidence also demonstrates enhanced student engagement, and improved patient-centred attitudes. Although it is important to note study designs were heterogenous and it is difficult to draw firm conclusions about the outcomes of digital medical education when patients and carers have been involved, particularly where it is unclear whether participants had lived experience or were scripted. Furthermore, patient and carer involvement was generally at a low level where there was no interaction with students, suggesting that digital technology has not yet driven the involvement of patients and carers much further beyond simulation. “Live” encounters with patients offered an opportunity to enhance students’ clinical and communication skills, although introduced additional barriers related to building rapport (between students and patients, and students and their supervisors) and issues with technology. This review does however demonstrate the potential benefits of involving patients and carers in medical education when teaching is delivered remotely.

The research in this area was limited in scope, with no studies directly capturing the perspective of the patients or carers involved in remote teaching. Thus, a balanced view of patient or carer participation, including any benefits and negative impacts for participants, and how educators might address these when organising teaching sessions, has not been obtained. In contrast, evidence from in-person medical education has identified a number of barriers to participation [12, 13]. Studies in this review identified potential challenges, including difficulty building rapport with patients, and between GP supervisors and students [27] and patients lacking access to connect with students [41]. However, no study has directly captured these issues from the viewpoint of patients’ and carers’ themselves. Without these key stakeholder perspectives, it remains unclear what additional barriers using remote teaching tools may introduce for those wishing to be involved.

### Links to previous research

Patients and carers have not been meaningfully involved in medical education when digital technologies have been used in teaching. This finding is not replicated by the growing body of literature reporting good examples of PPI in medical education [3]. The majority of studies in a recent systematic review described patients as educators and assessors, reflecting Level 4 of Towle’s taxonomy [3, 8]. One study included in our review involved patients with lived experience of the medical problems they portrayed, but their involvement was a scripted role, supposedly with the aim of standardising students’ learning experience [31]. This suggests there is still progress to be made to ensure patients and carers are equal partners in remote medical learning ensuring spaces for authentic interaction between students and patient about their lived experiences of illness and disease. Research from in-person teaching contexts has helpfully identified ways patients and carers wish to be involved, including wanting clear information before student encounters and a desire for their consent to be taken at each stage (e.g. may consent to student being present, but not taking a clinical examination) [12, 13]. With technology in educational activities significantly increasing in use since the beginning of the COVID-19 pandemic [7, 43, 44], the use of more interactive technologies (e.g. video-conferencing software) can provide students with valuable experiences interacting with and learning from patients and carers in real-time [7, 43]. Furthermore, without identifying the barriers associated with remote participation we risk further marginalising people already excluded [45], for example people with disabilities or who are homeless. Medical students would benefit from these viewpoints to better understand how to improve future healthcare service access in the era of digital health.

There was poor consistency in the use of terminology to describe patients and carers, including in studies where authentic patients or carers had been involved and where there had been no genuine patient or carer involvement (e.g. when actors were employed). Previous authors have highlighted the inconsistencies in meaning within and between common terms such as “virtual patient” or “simulated patient” [46, 47]. The diversity of meaning in these terms (and poor reporting of study methods) has implications for the replicability of medical education research evaluating the involvement of patients and/or carers. Going forward, researchers and educationalists may benefit from a new, standardised approach to terminology to ensure study replicability. For instance, Towle and colleagues clearly differentiate “patients” (who have a medical problem), from “simulated/ standardised” patients who role play symptoms and signs they do not actually have [8].

### Limitations

This was a rapid systematic review, conducted under time constraints and we acknowledge the potential to have excluded some relevant research. For example, articles published in foreign language and unpublished ongoing trials. We also acknowledge the inclusion of four studies where it remains unclear whether authentic patients or carers were involved, due to poor describing of methods. We decided to retain these studies as there was also no indication that patients or carers were not authentic. This raises an important issue whereby a lack of description inhibits a thorough assessment and replication of the study methods. We acknowledge that our PPI contributors were not involved in defining the research question, however their contribution to the review processes, and to our understanding of issues related to whether “authentic” patients were involved in educational activities or not, as described by study authors, was invaluable.

## Conclusions

Medical schools should ensure students’ learning is reflective of everyday healthcare practice during the COVID-19 pandemic and beyond, by incorporating PPI in remote learning. We have identified a variety of digital technologies used in medical teaching where patients and carers are involved. With the majority of studies in this review describing low levels of involvement, there is a need for medical schools to embrace recommendations to involve patients and carers as equal partners in the design, delivery and evaluation of medical curricular. Digital teaching sessions involving patients or carers were beneficial and found educationally valuable by students and educators, acceptable to students, and increased their engagement, patient-centred attitudes, clinical knowledge, and communication skills. Overall, quality of the studies included in this review was moderate to high; the results of studies of poor quality and those lacking clear descriptions of patients and carers should be viewed with caution. Future research should capture patients’ and carers’ views about their involvement in remote medical education (including any barriers and facilitators) to ensure future medical training is representative of local populations and to avoid digitally excluding marginalised groups.

## Supplementary Information

**Additional file 1.** Sample Search Strategy (Ovid MEDLINE).

## Data Availability

Our search strings used for the current study are available from the corresponding author on reasonable request.

## Abbreviations

GP: General Practitioner
MMAT: Mixed Methods Appraisal Tool
PPI: Patient and public involvement
SPIDER: Sample, Phenomenon of Interest, Design, Evaluation, Research
